# Surgical Treatment of Urinary Incontinence in the Woman

**Published:** 1893-12

**Authors:** 


Surgical Treatment of Urinary Incontinence in the Woman.—Gersuny and Zuckerkandl (*Internat. Klin. Rundschau*, No. 19, 1893). Incontinence of the urine in many women is by no means rare. As it is known, the slightest increase of abdominal pressure produced by coughing, laughing, or any other effort, causes the urine to escape. Therapeutic attempts have been made to remedy the weakness of the sphincter and the shortness of the urethra by cauterization of the external orifice of the canal. Three years ago, Gersuny proposed to twist the canal, and this operation has just been successfully performed by Zuckerkandl. The patient was a woman, fifty-four years of age. Examination showed the bladder to be in normal condition. She was afflicted, however, with the variety of incontinence just described, and had been submitted to various modes of treatment without favorable result. Zuckerkandl made an oval incision around the urethral orifice, and separated the walls of the canal up to the bladder. By then twisting the urethra in the direction of the hands of a clock, he obtained a rotation of 360°. The external orifice was then fixed in this new position with a row of stitches, a sound was left in place for two days, and after fourteen days' rest the cure was complete, and the patient could easily retain her urine. It is well to remember that the first patient operated on by Gersuny in this way, three years ago, has never again been troubled by incontinence, but has, on the contrary, to make an effort to pass water.—*New York Therapeutic Review.*

